# A Case of Non-Small Cell Lung Cancer with Possible “Disease Flare” on Nivolumab Treatment

**DOI:** 10.1155/2016/1075641

**Published:** 2016-12-27

**Authors:** Shotaro Chubachi, Hiroyuki Yasuda, Hidehiro Irie, Koichi Fukunaga, Katsuhiko Naoki, Kenzo Soejima, Tomoko Betsuyaku

**Affiliations:** ^1^Division of Pulmonary Medicine, Department of Medicine, Keio University School of Medicine, Shinjuku-ku, Tokyo, Japan; ^2^Cancer Center, Keio University School of Medicine, Shinjuku-ku, Tokyo, Japan; ^3^Clinical and Translational Research Center, Keio University School of Medicine, Shinjuku-ku, Tokyo, Japan

## Abstract

*Background*. Recent clinical trials proven the clinically significant efficacy and tolerability of nivolumab, a programmed death 1 (PD-1) inhibitor, in previously treated patients with non-small cell lung cancer (NSCLC).* Case Presentation*. Here, we describe the case of a patient who experienced possible “disease flare” immediately after initiation of nivolumab treatment. A 54-year-old man was diagnosed with Stage IIB (T2N1M0) lung adenocarcinoma. After 7 years from recurrence, 10th line chemotherapy, nivolumab, was initiated. Six weeks later, after 3 cycles of nivolumab treatment, rapid lung cancer progression was observed with an increase in the size of the primary lesion, multiple novel nodules on both lungs, and multiple novel brain metastases.* Conclusion*. We believe that physicians should be made aware that, in a subset of NSCLC patients, disease flare might occur on nivolumab treatment.

## 1. Introduction

Immune checkpoint inhibitors such as nivolumab are promising new therapies for advanced cancers [1]. Here, we describe the case of a patient who experienced possible “disease flare” immediately after initiation of nivolumab treatment.

## 2. Case Presentation

A 54-year-old man was diagnosed with Stage IIB (T2N1M0) lung adenocarcinoma. He underwent a right upper lobectomy and adjuvant chest irradiation therapy. However, 10 months after treatment, a follow-up computed tomography (CT) scan revealed multiple lung tumors. A biopsy of the right lung tumor was histologically confirmed as adenocarcinoma, matching the primary resected tumor. Molecular profiling of the relapsed tumor clarified that the tumor harbored epidermal growth factor receptor (*EGFR*) exon 19 deletion. Thereafter, he underwent treatment with multiple lines of chemotherapy including EGFR tyrosine kinase inhibitors (EGFR-TKIs), including gefitinib, erlotinib, and afatinib, and cytotoxic agents. During this treatment period, a second biopsy was performed and an additional* EGFR* T790M mutation was confirmed.

**Figure 1:**
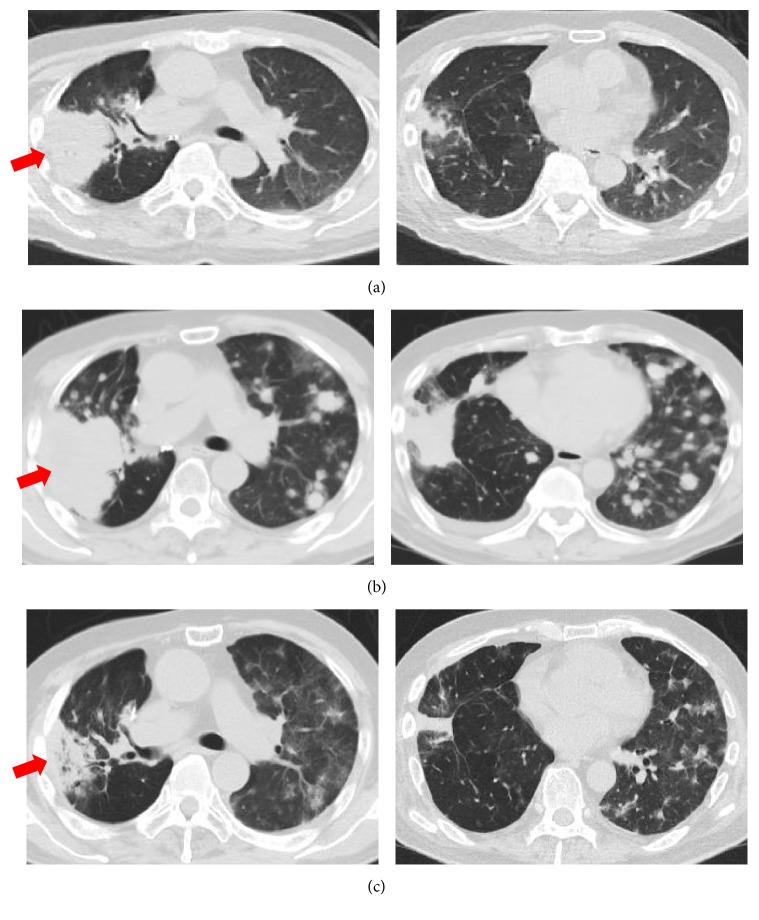
CT scan of the chest (a) before nivolumab treatment, (b) after 3 cycles of nivolumab treatment, and (c) osimertinib day 14.

The clinical course of the disease was indolent until he was initiated on nivolumab treatment. The latest chemotherapy regimen before commencing nivolumab was gemcitabine (1000 mg/m^2^ on days 1, 8, and 15 of a 4-week cycle) plus vinorelbine (25 mg/m^2^ on days 1, 8, and 15 of a 4-week cycle), which was administered from December 2015. However, after 2 cycles, the treatment was discontinued because the patient was admitted to another hospital with a right femoral bone fracture. However, there was no evidence of lung cancer metastasis to the bone. After rehabilitation, the patient revisited our hospital to resume chemotherapy for lung cancer. At this time, disease progression was observed with an increase in the primary tumor size (Figure 1(a) arrow) and multiple brain metastases, but disease progression was still considered indolent.

As a 10th line chemotherapy, nivolumab (3 mg/kg, every 2 weeks) was initiated on March 2016. The patient's Eastern Cooperative Oncology Group (ECOG) performance status was 1 before and after nivolumab treatment. Six weeks later, after 3 cycles of nivolumab treatment, a whole body CT and magnetic resonance imaging of the head were performed to evaluate treatment response. However, rapid lung cancer progression was observed with an increase the size of the primary lesion located in the right upper lobe (Figure 1(b) arrow), multiple novel nodules on both lungs (Figure 1(b)), and multiple novel brain metastases. In addition, his serum carcinoembryonic antigen (CEA) levels had increased markedly and rapidly from 360.4 ng/mL to 1316.0 ng/mL, over approximately 8 weeks. He was urgently hospitalized because of right-side paralysis caused by the brain metastases. Following the approval of osimertinib for use in Japan, we commenced treatment with the drug immediately. After 14 days of osimertinib initiation, a whole-body CT revealed drastic shrinkage of the primary lung tumor (Figure 1(c) arrow) and multiple metastatic lung lesions (Figure 1(c)).

## 3. Discussion

Nivolumab, a fully humanized immunoglobulin G4 anti-programmed death 1 (PD-1) antibody, has demonstrated clinically meaningful efficacy and a manageable safety profile in patients with previously treated advanced NSCLC [2, 3]. Following approval of nivolumab for these patients, the clinical use of nivolumab in Japan has become widespread. However, disease progression or pseudoprogression during nivolumab treatment has been reported [4]. However, in the present case, serum CEA levels were dramatically elevated following nivolumab initiation, whereas osimertinib treatment drastically shrank both the primary and multiple metastatic lung lesions. These findings indicate that the nivolumab-associated progression was not “pseudo” but “real.” Considering that the disease followed an indolent course until nivolumab treatment and that the disease progression was so drastic after nivolumab treatment, we believe the progression represented a “disease flare.” Disease flares after the discontinuation of EGFR-TKIs have been reported and were clinically defined as accelerated disease progression [5, 6]. To our knowledge, this is the first report of a possible “disease flare” during nivolumab treatment.

PDL1 expression level was reported to be a predictive marker of response in immune checkpoint inhibitors, such as nivolumab and pembrolizumab [2, 7]. Recently,* EGFR* mutations were reported to be a possible unfavorable marker of response in these agents [8]. However, there is no marker to predict the “disease flare” induced by nivolumab until today.

## 4. Conclusion

The mechanism underlying this potential disease flare is unknown and further studies would be needed to investigate a putative mechanism. In addition, the frequency of such disease flairs would require accumulation of further cases; therefore, we believe that physicians should be made aware of the potential of nivolumab to induce disease flair in patients with previously treated NSCLC.

## Abbreviations

EGFR: Epidermal growth factor receptor
PD-1: Programmed death 1
NSCLC: Non-small cell lung cancer.
